# A p.Arg499His mutation in *SPAST* is associated with infantile-onset complicated spastic paraplegia: a case report and review of the literature

**DOI:** 10.1186/s12883-021-02478-0

**Published:** 2021-11-09

**Authors:** Haitian Nan, Hiroshi Shiraku, Tomoko Mizuno, Yoshihisa Takiyama

**Affiliations:** 1 Department of Neurology, Graduate School of Medical Sciences, University of Yamanashi, Yamanashi, 409-3898 Japan; 2 Department of Pediatrics, JA Toride Medical Center, Ibaraki, 302-0022 Japan; 3 Department of Pediatrics, Tokyo Medical and Dental University, Tokyo, 113-8510 Japan

**Keywords:** Hereditary spastic paraplegia, SPG4, Complicated form, P.Arg499His, Case report

## Abstract

**Background:**

Spastic paraplegia type 4 (SPG4) is caused by mutations in the *SPAST* gene, is the most common form of autosomal-dominant pure hereditary spastic paraplegias (HSP), and is rarely associated with a complicated form that includes ataxia, epilepsy, and cognitive decline. To date, the genotype-phenotype correlation has not been substantially established for *SPAST* mutations.

**Case presentation:**

We present a Japanese patient with infantile-onset HSP and a complex form with coexisting ataxia and epilepsy. The sequencing of *SPAST* revealed a de novo c.1496G > A (p.R499H) mutation. A review of the literature revealed 16 additional patients with p.R499H mutations in *SPAST* associated with an early-onset complicated form of HSP. We found that the complicated phenotype of patients with p.Arg499His mutations could be mainly divided into three subgroups: (1) infantile-onset ascending hereditary spastic paralysis, (2) HSP with severe dystonia, and (3) HSP with cognitive impairment. Moreover, the c.1496G > A mutation in *SPAST* may occur as a de novo variant at noticeably high rates.

**Conclusion:**

We reviewed the clinical features of the patients reported in the literature with the p.Arg499His mutation in *SPAST* and described the case of a Japanese patient with this mutation presenting a new complicated form. Accumulating evidence suggests a possible association between infantile-onset complicated HSP and the p.Arg499His mutation in *SPAST.* The findings of this study may expand the clinical spectrum of the p.Arg499His mutation in *SPAST* and provide an opportunity to further study the genotype-phenotype correlation of SPG4.

**Supplementary Information:**

The online version contains supplementary material available at 10.1186/s12883-021-02478-0.

## Background

Hereditary spastic paraplegias (HSPs) are clinically and genetically heterogeneous neurodegenerative disorders characterized by progressive weakness and spasticity in the lower limbs due to pyramidal tract dysfunction [1]. HSPs can be inherited in an autosomal-dominant (AD), autosomal-recessive (AR), X-linked, or mitochondrial manner [2]. An isolated pyramidal syndrome affecting predominantly in the lower limbs with or without vibration sense impairment and urinary urgency defines pure HSP, whereas complicated HSP presents a more complex clinical picture with additional neurological findings, including ataxia, epilepsy, and cognitive decline [1, 2]. AR-HSP is usually a complicated form, contrary to AD-HSP, which is mostly a pure form [3].

Spastic paraplegia type 4 (SPG4) is due to heterozygous mutations in the *SPAST* gene and is the most frequent cause of both familial and sporadic HSP [1]. SPG4 in most cases is considered a pure HSP. SPG4 is rarely associated with additional neurological signs [4]. Here, we present the case of a Japanese patient with a complex form of infantile-onset HSP with coexisting ataxia and epilepsy. *SPAST* sequencing revealed a de novo c.1496G > A (p.R499H) mutation. A review of the literature revealed 16 additional patients with p.R499H mutations in *SPAST* associated with early-onset complicated forms of HSP.

## Case presentation

**Fig. 1:**
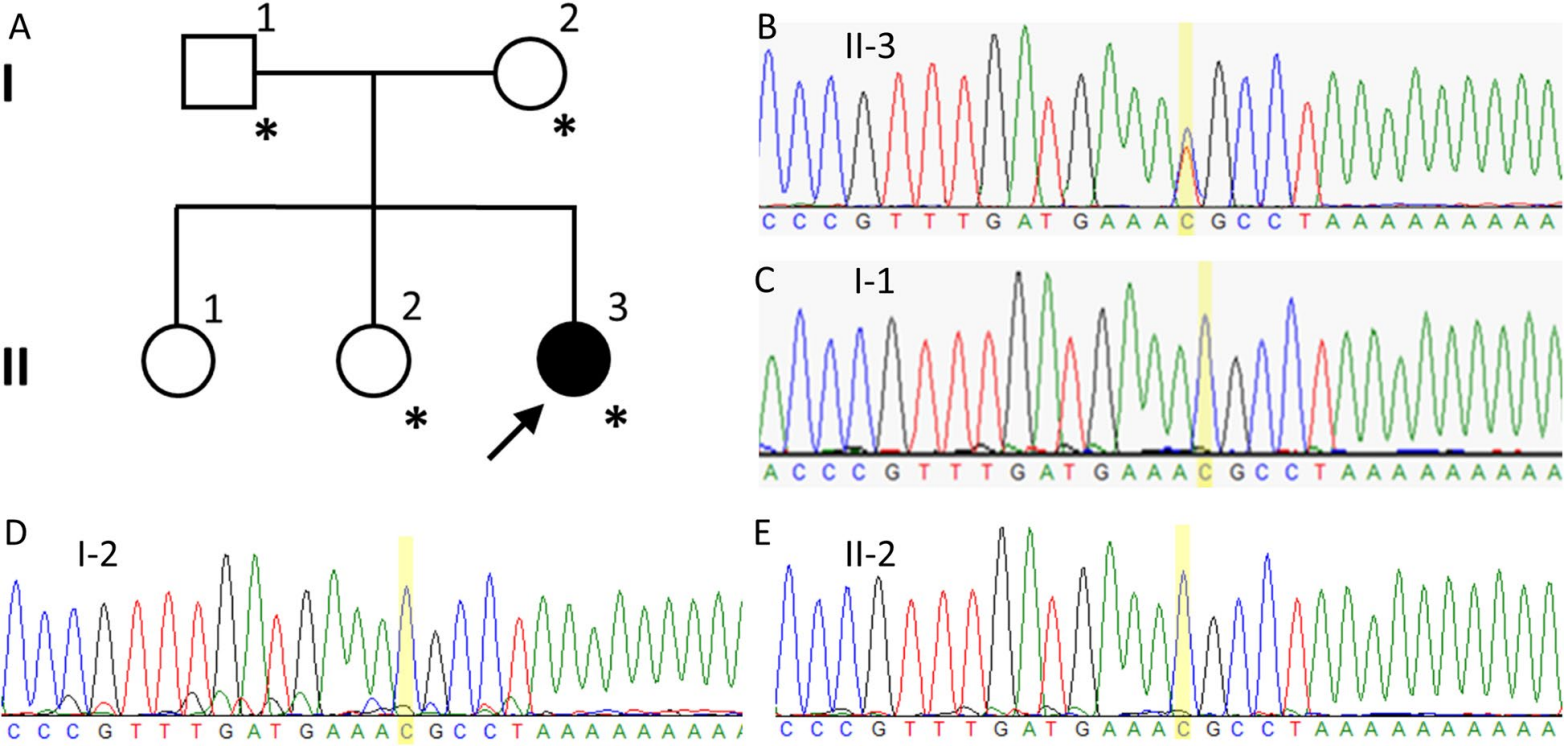
**A** Pedigree of the present family. The proband is indicated (arrow). Squares indicate men, circles women, shaded (black) symbols individuals with symptoms of complicated HSP, and unshaded ones individuals without any symptoms. Individuals evaluated both clinically and genetically are denoted by asterisks. **B** Sanger sequencing revealed the c.1496G > A mutation in *SPAST* was in a heterozygous state in the proband. The c.1496 nucleotide is shaded yellow. **C** The c.1496G > A mutation in *SPAST* was not detected in the proband’s father. The c.1496 nucleotide is shaded yellow. **D** The c.1496G > A mutation in *SPAST* was not detected in the proband’s mother. The c.1496 nucleotide is shaded yellow. **E** The c.1496G > A mutation in *SPAST* was not detected in the proband’s younger sister. The c.1496 nucleotide is shaded yellow

A 4-year-old girl (Fig. 1A, II-3) was the third of three siblings born to healthy and unrelated parents. Her 8-year-old and 5-year-old sisters (Fig. 1A II-1 and II-2) were unaffected. She was born by vaginal delivery after an uneventful pregnancy. Her parents initially became concerned when she could not crawl on her hands and knees or stand unassisted by 17 months of age. She could lift her head by 4 months, roll over by 6 months, sit up unsupported by 10 months, crawl on her belly by 11 months, stand with assistance by 18 months, and walk with assistance by 22 months. She could not walk independently until now. A neurological examination at 17 months of age revealed slight muscle weakness in the distal lower extremities and exaggerated deep tendon reflexes in the lower limbs. She started rehabilitation therapy at that point. At 2 years and 3 months, she presented a scissor gait when walking with assistance. At 2 years and 5 months, truncal ataxia was detected. She was intellectually normal, and no nystagmus, intention tremor, dysmetria, or speech impairment was detected. At 3 years and 3 months, the Kyoto Scale of Psychological Development (KSPD) test revealed no cognitive developmental delay, with the following scores: Postural-Motor functions, 26; Cognitive-Adaptive functions, 96; and Language-Social functions, 81. A nerve conduction study revealed nothing abnormal. Metabolic and routine blood investigations were unremarkable.

At 3 years and 5 months, she had an unprovoked epileptic seizure with forced rightward eye deviation. Although acute symptomatic seizures did not occur after that seizure, nonsymptomatic electroencephalogram (EEG) abnormalities with centroparietal spikes were repeatedly recorded. Epilepsy was diagnosed, and she was started on levetiracetam, which she continues to take. The abnormal EEG wave disappeared after antiepileptic treatment, and has been seizure-free ever since.

The motor symptoms of the patient progressed slowly, and her gait became increasingly slow and spastic over time. On neurological examination at age 4, she presented slight paresis, increased muscle reflexes, and increased muscle tone and stiffness in all four limbs. Knee and ankle clonus and bilateral Babinski signs were detected. Truncal ataxia, intention tremor, and a staggering gait were also detected. There were no speech or eye movement abnormalities. Magnetic resonance imaging (MRI) of the brain and spine performed at ages 2, 3, and 4 revealed no abnormalities.

We carried out whole-exome sequencing of genomic DNA from the patient. Genomic DNA was extracted from peripheral blood. Exome capture was performed with a SureSelect Human All Exon V6 + UTR (89 Mb) Kit (Agilent Technologies, Santa Clara, CA, USA). Paired-end sequencing was carried out on a HiSeq2500 (Illumina, San Diego, CA, USA) using a HiSeq SBS Kit V4 (Illumina), which generated 100-bp reads. The reference databases utilized included hg38 (GRCh38) (http://genome.ucsc.edu), HGMD (https://portal.biobase-international.com), gnomAD (http://gnomad.broadinstitute.org), and dbSNP (https://www.ncbi.nlm.nih.gov/SNP). We examined variants of 250 genes known to be responsible for or associated with spinocerebellar ataxia, Charcot-Marie-Tooth disease, or HSP (Additional file 1). Through this analysis, we identified a heterozygous missense mutation (c.1496G > A, p.Arg499His) in exon 13 of the *SPAST* gene in the patient and ruled out the possibility of other causative genes. We then examined exon 13 of the *SPAST* gene in the patient as well as the patient’s father (Fig. 1A, I-1), mother (Fig. 1A, I-2), and younger sister (Fig. 1A, II-2) by Sanger sequencing. On Sanger sequencing, we reconfirmed the p.R499H mutation in exon 13 of the *SPAST* gene, which was in a heterozygous state in the patient (Fig. 1B). On the other hand, the patient’s parents and younger sister did not have the mutation (Fig. 1C, D, E). In this family, the patient harbored a mutation that was absent in her parents and her healthy sibling. Her oldest sister was also healthy. These observations suggest that the mutation occurred de novo in the patient.

## Discussion and conclusion

The p.R499H mutation in the *SPAST* gene has been reported as a disease-causing mutation in many previous studies [5–20]. This mutation is located in the AAA ATPase cassette of spastin (from amino acids 342 to 616), which is crucial for microtubule-severing activity [4]. The clinical features of patients with the p.Arg499His mutation reported in the literature are briefly summarized in Table 1.

**Table 1:** Brief clinical features of patients with the c.1496G > A p.Arg499His mutation in *SPAST* reported in the literature

Patient	Inheritance mode	Sex	Age at onset	Phenotype	Country
1	de novo	Male	14 months	Infantile-onset ascending hereditary spastic paralysis, anarthria	Japan [5]
2	de novo	Male	18 months	Pure HSP	Greece [6]
3	de novo	Female	20 months	HSP, intellectual disability	Canada [7]
4	de novo	Female	12 months	HSP, early expressive language delay	Canada [7]
5	de novo	Female	6 years	Pure HSP	Netherlands [8]
6	de novo	Female	< 2 years	HSP, intellectual disability, loss of speech, severe dysphagia, epilepsy: three generalized seizures with spontaneous recovery	Netherlands [8]
7	de novo	Female	1 month	HSP, intellectual disability, loss of speech, severe dysphagia, epilepsy: febrile seizure at ages 1 and 3 y, scoliosis, urinary and fecal incontinence	Netherlands [8]
8	de novo	Male	1 week	HSP, severe dystonia, loss of speech, severe dysphagia, severe scoliosis, urinary incontinence	Netherlands [8]
9	de novo	Male	< 2 years	HSP, intellectual disability, loss of speech, dysarthria	Netherlands [8]
10	de novo	Female	< 2 years	HSP, loss of speech, dysarthria, severe dysphagia	Netherlands [8]
11	de novo	Unknown	6 years	Pure HSP	Italy [9]
12	Autosomal dominant	Unknown	< 10 years	HSP, trunk-ataxia	Germany [10]
13	de novo	Male	26 month	HSP, intellectual disability	Japan [11]
14	de novo	Female	< 2 years	HSP, epilepsy, dysarthria, dysphagia, tongue fasciculation	Japan [12]
15	de novo	Female	< 2 years	Infantile-onset ascending spastic paralysis, dysphagia, severe dysarthria, lower limb deep sensory loss, slow saccadic eye movements	Brazil [13]
16	de novo	Male	Infancy	HSP, generalized dystonia	Germany [14]
17	de novo	Female	Infancy	HSP, generalized dystonia	Germany [14]
18	de novo	Male	< 8 years	Pure HSP	China [15]
19	Autosomal dominant	Male	1 year	Pure HSP	Korea [16]
20	Unknown	Unknown	Unknown	HSP, dystonia, white matter abnormality on MRI	Netherlands [17]
21	de novo	Male	Childhood	Pure HSP	France [18]
22	Autosomal dominant	Female	1 year	Pure HSP	Korea [19]
23	Autosomal dominant	Male	< 4 years	HSP, intellectual disability	Japan [20]
24	de novo	Female	17 months	HSP, cerebella ataxia, epilepsy	This study

Including our patient, clinical information is available for 24 patients from different parts of the world. Among them, seventeen patients presented a complicated phenotype, while only 7 were reported to have a pure type of HSP. Admittedly, SPG4 patients with complicated phenotypes are more likely to be reported by clinicians, and our review is strictly not epidemiological. However, it is noteworthy that patients with p.Arg499His mutations are significantly more frequently associated with complicated phenotypes than other mutations in *SPAST*. Additionally, the patients with this mutation almost invariably also suffered from a more severe type of spastic paraplegia with onset in the first or second year of life. The complicated phenotypes of patients with p.Arg499His mutations can be primarily divided into three subgroups: (1) infantile-onset ascending hereditary spastic paralysis (Patients 1, 6, 7, 9, 10, 14, and 15). These patients all presented very-early-onset (< 2 years old) spastic paraplegia with an ascending phenotype with bulbar involvement mostly occurring during their first decades. (2) Patients 8, 16, 17, and 20 presented early-onset spastic paraplegia with dystonia. (3) Patients 3, 4, 13, and 23 presented early-onset spastic paraplegia with cognitive impairment. Apart from our patient, only one patient (Patient 12) was reported to have associated trunk ataxia in the literature [10]. No further information was provided for that patient; however, both patients presented trunk ataxia as the key symptom of cerebellar dysfunction. Three of the 23 patients (Patients 6, 7, and 14) reported in the literature also had seizures, while Patients 6 and 7 also had an intellectual disability. In contrast, in our patient, cognitive ability was preserved, as evaluated by the KSPD test. Interestingly, most of the patients (19 out of 24) had a de novo mutation, and only 4 were reported to have a family history. This is partly because most patients with the p.Arg499His mutation in *SPAST* presented a very severe phenotype with a very early onset age. Moreover, this mutation is located in the AAA domain of the *SPAST* gene, where mutations are clustered [10]. Nevertheless, it is worth noting that the c.1496G > A mutation in *SPAST* may occur as a de novo variant at noticeably high rates.

We have no explanation for the fact that the p.Arg499His mutation in *SPAST* can lead to pure HSP as well as to the highly complex phenotypes presented here. The most recognized genetic modifier, c.131C > T (p.Ser44Leu), in *SPAST* was negative in our patient [4]. There might be some unknown genetic etiology or environmental modifiers underlying the complicated symptoms that could not be identified with the present methods. To date, the genotype-phenotype correlation has not been substantially established for *SPAST* mutations [4]. However, accumulating evidence suggests that the p.Arg499His mutation in *SPAST* is associated with severe infantile-onset complicated HSP. Whether the etiology of SPG4 is haploinsufficiency or toxic gain-of-function properties of the mutant spastin proteins remains controversial. The evidence gathered here is not indicative of haploinsufficiency as the cause of the disease since certain mutations in *SPAST* are more dangerous than others, which would support a toxic gain-of-function etiology, at least in certain missense mutations. Future studies will hopefully allow the further delineation of the complex phenotype associated with the p.Arg499His mutation and the understanding of the underlying molecular mechanisms.

In conclusion, we recognized a complex SPG4 phenotype with infantile-onset severe spastic paraplegia complicated by cerebellar ataxia and epilepsy. This specific phenotype has not previously been reported in the clinical spectrum of SPG4 patients with p.Arg499His mutations. Currently, our patient may still be too young for final phenotyping and thus will need to be followed carefully. A review of the literature revealed Arg499His may occur as a de novo *v*ariant at noticeably high rates. The patient we reported here, together with the cases previously reported in the literature, suggests an association between infantile-onset complicated HSP and the p.Arg499His mutation in *SPAST.* This study may provide an opportunity to further study the genotype-phenotype correlation of SPG4 and shed light on clinicians performing genetic testing for patients with early-onset complicated HSP.

## Supplementary Information

**Additional file 1: Supplementary Table 1.** Genes known to be responsible for or associated with spinocerebellar ataxia, Charcot-Marie-Tooth disease, or HSP.

**Additional file 2.** CARE-checklist.pdf.

## Data Availability

All data generated or analyzed during this study are included in this published article.

## Abbreviations

HSP: Hereditary spastic paraplegias
AD: Autosomal dominant
AR: Autosomal recessive
MRI: Magnetic resonance imaging
SPG4: Spastic paraplegia type 4
KSPD: Kyoto Scale of Psychological Development
EEG: Electroencephalography
