# Supplementary material for: A p.Arg499His mutation in SPAST is associated with infantile-onset complicated spastic paraplegia: a case report and review of the literature
Source: BMC Neurol. 2021 Nov 9;21:439. doi: 10.1186/s12883-021-02478-0 (PMC8576993; doi:10.1186/s12883-021-02478-0)
Supplement: Supplementary file 1 — Additional file 1: Supplementary Table 1. Genes known to be responsible for or associated with spinocerebellar ataxia, Charcot-Marie-Tooth disease, or HSP. [file 12883_2021_2478_MOESM1_ESM.docx]

Supplementary Table 1. Genes known to be responsible for or associated with spinocerebellar ataxia, Charcot-Marie-Tooth disease, or HSP.

*CWF19L1 COA7 ELOVL4 ATXN10 PPP2R2B WWOX KCNC3 PRKCG ITPR1 CNTN4 IFRD1 KCND3 ATXN2 TMEM240 PDYN EEF2 FGF14 AFG3L2 ATXN3 BEAN1 TGM6 NOP56 DAB1 ELOVL5 CACNA1G STUB1 SPTBN2 CACNA1A ATXN8OS TDP1 SLC52A2 MME SYT14 GRM1 ANO10 SYNE1 COQ4 TWNK TTBK2 ATP2B3 COQ8A CCDC88C FAT2 PLD3 KIF26B FAT1 EP300 SNX14 SPG7 GJB1 CYP7B1 TPP1 ATXN8 TDP2 GRID2 TTC19 GBA KCNJ10 SCA25 ATXN7 ADH1C SCA30 ATXN1 TBP SCA32 NEFL TTPA CAPN1 ATN1 SCA4 SLC4A1 MAPT MYADM GLUD2 16q22 7q31 11q12 2p15-p21 4q34 7q32 11q21 TRPC3 PUM1 SLC1A3 ATL1 SPAST NIPA1 KIAA0196 ALDH18A1 KIF5A RTN2 HSPD1 BSCL2 REEP1 ZFYVE27 SLC33A1 REEP2 CPT1C SPG11 ZFYVE26 ERLIN2 SPG20 SPG21 B4GALNT1 DDHD1 FA2H PNPLA6 C19orf12 GJC2 NT5C2 GBA2 AP4B1 AP5Z1 TECPR2 AP4M1 AP4E1 AP4S1 VPS37A DDHD2 C12orf65 CYP2U1 TFG KIF1C USP8 WDR48 ARL6IP1 ERLIN1 AMPD2 ENTPD1 ARSI PGAP1 FLRT1 RAB3GAP2 MARS ZFR IBA57 MAG MT-CO3 MT-TI MT-ND4 MT-ATP6 L1CAM PLP SLC16A2 BICD2 CHS IFIH1 CCT5 FAM134B ALS2 EXOSC3 GAD1 HACE1 LYST SACS AARS ABHD12 AIFM1 ARHGEF10 ARSA ASAH1 COX6A1 CTDP1 DCAF8 DGAT2 DHH DHT DNAJB2 DNAJC3 DNM2 DRP2 DYNC1H1 EGR2 EMILIN1 FBLN5 FGD4 FIG4 GALC GAN GARS GDAP GDAP1 GJB3 GNB4 HARS HINT1 HK1 HOXD10 HSPB1 HSPB8 IGHMBP2 INF2 KARS SLC12A6 KIF1B LITAF LMNA LRSAM1 MED25 MFN2 MORC2 MPZ MTMR2 NAGLU NDRG1 NEFH PDK3 PEX7 PHYH PLA2G6 PLEKHG5 PMM2 PMP22 PRPS1 PRX RAB7 SBF1 SBF2 SCYL1 SH3TC2 SLC25A46 SOX10 SPTLC1 SPTLC2 SPTLC3 SURF1 TRIM2 TRPV4 TUBB3 VCP YARS KIF1A UBAP1 HPDL SELENOI PCYT2 KCNA2 KIDINS220 UCHL1 ATP13A2 FARS2 KLC2 SOD1 ACO2 RNF170 DESI1 WASHC5 MTTV SYNE3 SPTBN5*
